# Hand Washing Practices Among Emergency Medical Services Providers

**DOI:** 10.5811/westjem.2015.7.25917

**Published:** 2015-10-20

**Authors:** Joshua Bucher, Colleen Donovan, Pamela Ohman-Strickland, Jonathan McCoy

**Affiliations:** * Rutgers Robert Wood Johnson Medical School, Department of Emergency Medicine, New Brunswick, New Jersey; † Rutgers School of Public Health, New Brunswick, New Jersey

## Abstract

**Introduction:**

Hand hygiene is an important component of infection control efforts. Our primary and secondary goals were to determine the reported rates of hand washing and stethoscope cleaning in emergency medical services (EMS) workers, respectively.

**Methods:**

We designed a survey about hand hygiene practices. The survey was distributed to various national EMS organizations through e-mail. Descriptive statistics were calculated for survey items (responses on a Likert scale) and subpopulations of survey respondents to identify relationships between variables. We used analysis of variance to test differences in means between the subgroups.

**Results:**

There were 1,494 responses. Overall, reported hand hygiene practices were poor among pre-hospital providers in all clinical situations. Women reported that they washed their hands more frequently than men overall, although the differences were unlikely to be clinically significant. Hygiene after invasive procedures was reported to be poor. The presence of available hand sanitizer in the ambulance did not improve reported hygiene rates but improved reported rates of cleaning the stethoscope (absolute difference 0.4, p=0.0003). Providers who brought their own sanitizer were more likely to clean their hands.

**Conclusion:**

Reported hand hygiene is poor amongst pre-hospital providers. There is a need for future intervention to improve reported performance in pre-hospital provider hand washing.

## INTRODUCTION

Healthcare worker compliance with hand hygiene remains a pervasive problem in medicine. Physicians have notoriously poor compliance.^1^–^3^ The lack of hand hygiene compliance results in transmission of community-acquired and hospital-acquired microorganisms between both patients and providers, which can lead to nosocomial infections. Unfortunately, compliance remains stubbornly low despite efforts to change. While poor hand hygiene is prevalent in the hospital, these behaviors may also be similar among pre-hospital providers. However, hygienic behavior has been infrequently studied in the pre-hospital healthcare worker population despite the fact that it is a key part of the healthcare system

Pre-hospital emergency care inherently increases the risks of spreading infection. Pre-hospital providers often have contact with multiple patients per day, with varying conditions and states of immunocompetence. Hand washing compliance among pre-hospital providers has not been studied in the United States. Emergency medical technicians and paramedics frequently come into contact with patients in their homes or other social environments. Their unique role and practice environment could permit the transmission of a high burden of nosocomial inocula to patients or introduce community-acquired infections into the hospital.

A 2011 study identified that patients who were treated and transported by Advanced Life Support (ALS) paramedics had a higher rate of nosocomial infection than patients not transported by ALS. While this study was a retrospective review of admitted patients, ALS transport was associated with an odds ratio (OR) of 1.42 for suffering from a nosocomial infection, compared to patients with community-acquired infections.^4^ Admittedly, there may be a bias that ALS transported more ill patients who may be at risk of nosocomial infection at baseline.

Since emergency medical services (EMS) providers also operate the ambulance, there are many places for ambulance and personal equipment to become contaminated. One German study found that the highest areas of contamination were blood pressure cuffs, stethoscopes and the hand-washing area (not found on U.S., ambulances).^5^ Disposing of multi-use items may quickly prove cost prohibitive when considering the high volume of emergency service calls in many systems.

Moreover, there is room for improvement within EMS providers’ hand hygiene practices as well as ambulance and equipment cleaning. Merlin et al. found that 32% (16/50) of the stethoscopes used in a single EMS agency (providing both basic and ALS) grew methicillin-resistant *Staphylococcus aureus* (MRSA), and that 32% (16/50) of employees did not know the last time they had cleaned their stethoscopes. It also found that time from last cleaning was significantly associated with an increased chance of culturing MRSA (OR 1.86).^6^

Studying EMS worker hand hygiene practices is important for several reasons. Determining the rates of pre-hospital hand hygiene will help medical directors and educators develop policies to increase awareness and identify shortcomings in pre-hospital hygiene. It may also help identify obstacles to hand hygiene that prevent EMS providers from cleaning their hands adequately. It could help reduce transmission of microorganisms between patients and EMS providers and prevent contamination of equipment that patients frequently come into contact with, such as backboards, cervical collars, blood pressure cuffs, stethoscopes and other patient transport devices.

Our primary goal is to determine the rates of hand hygiene practices in a broad spectrum of EMS healthcare providers across a variety of clinical situations. Our secondary goal is to show the rates of providers’ stethoscope cleaning. We expect our results to lead to further investigation of obstacles and potential solutions to the problem of infection control in the EMS setting.

## METHODS

We designed an online survey distributed to EMS providers with questions about demographics and hand hygiene practices. The survey was sent to various organizations through a standardized e-mail that explained the purpose of the study, our goals, the length of the study and a link to the online survey (Appendix A). We used a convenience sample of EMS providers across a range of organizations to achieve a varied group (Appendix B).

Since the survey was sent out to large organizations for them to send to their distribution lists on a voluntary basis, we are unable to calculate a response rate.

The survey was designed to inquire about hand hygiene practices during different points of an EMS run, including prior to arrival at the scene, during patient treatment and after patient transfer. It was reviewed by all study members prior to distribution. The survey was screened by several EMS healthcare providers prior to generalized distribution in order to assess for appropriateness. Their feedback was incorporated into the survey in terms of the question inclusion, design and answer choices.

The survey received institutional review board approval at our institution.

We calculated frequencies as well as means with standard deviations (SDs) for each item on the survey. Means for hygiene items (responses ranged on a Likert scale from 1=Never to 5=Every time) were calculated for each subgroup. We defined subgroups by gender, age, level of training, whether paid/volunteer/both, years of experience, hygiene training, Body Substance Isolation (BSI) training, whether or not there was sanitizer in the ambulance or ambulance station, and the provider having his or her own sanitizer. Analysis of variance (ANOVA) was used to test differences in means between the subgroups. We used multivariate analysis of variance (MANOVA) to examine whether the cleaning responses were collectively different by subgroup. In these models, we included only a single predictor at a time.

Multivariable linear models were used to examine which predictors uniquely contributed to each response. In particular, we used a backwards stepwise regression model with all variables, with p-values greater than 0.05 eliminated from the model. Note that due to the large sample size and a desire to avoid over-fitting, we chose to use a strict alpha value (0.05) for the exit criteria. Also, note that in order to maintain comparability between models, only observations with data for all participant characteristics were included in these linear models.

We used proportional odds modeling as a means to determine the correct predictors for a multivariable model. Since results were similar to those obtained from the standard linear modeling, results are further described.

Physicians who responded were all EMS physicians who provided some pre-hospital supervision, education and administrative duties of the organization. The exact amount of pre-hospital patient contact was not investigated.

## RESULTS

**Figure 1:**
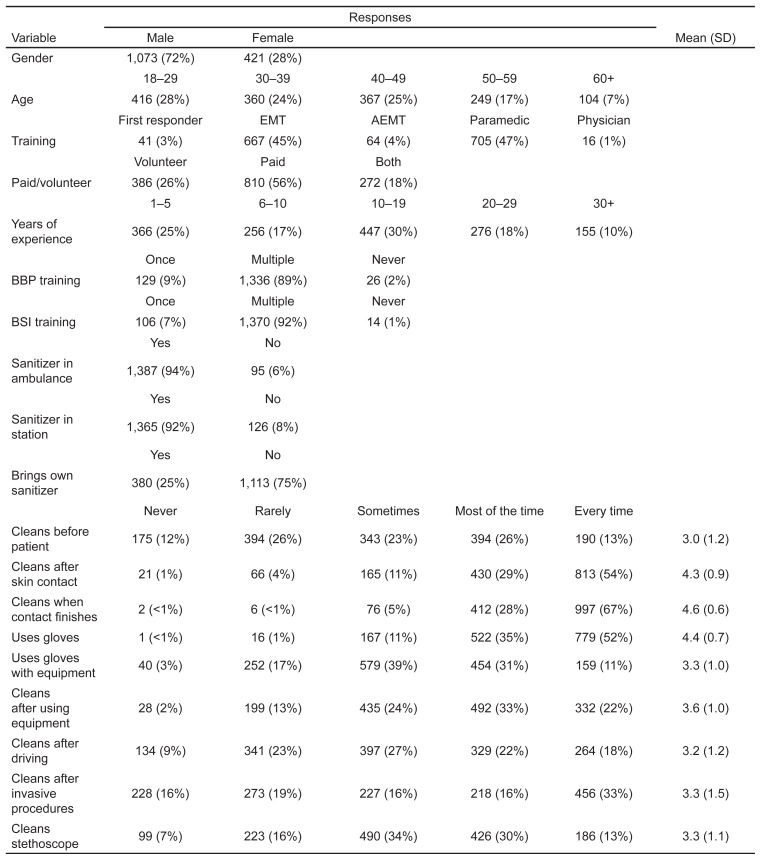

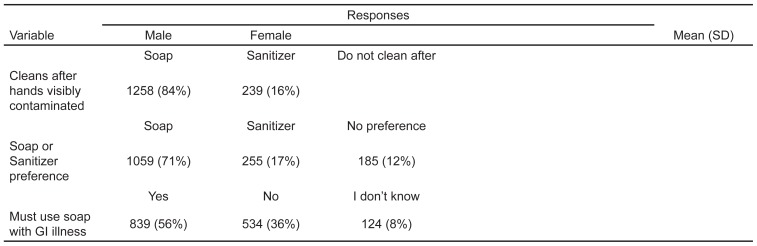
Frequencies (percentages) and means (standard deviations, SDs) for participant characteristics and responses of emergency medical services personnel in hand hygiene study. *EMT*, emergency medical technician; *AEMT*, advanced emergency medical technician; *BSI*, body substance isolation; *BBP,* blood borne pathogens

**Figure 2:**
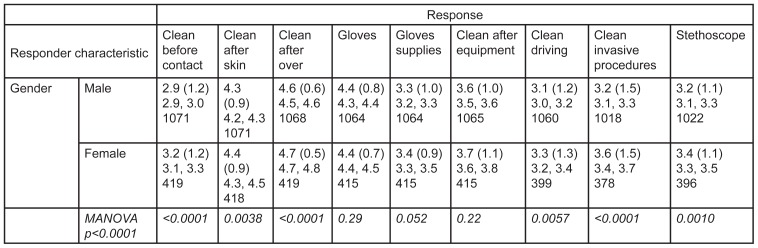

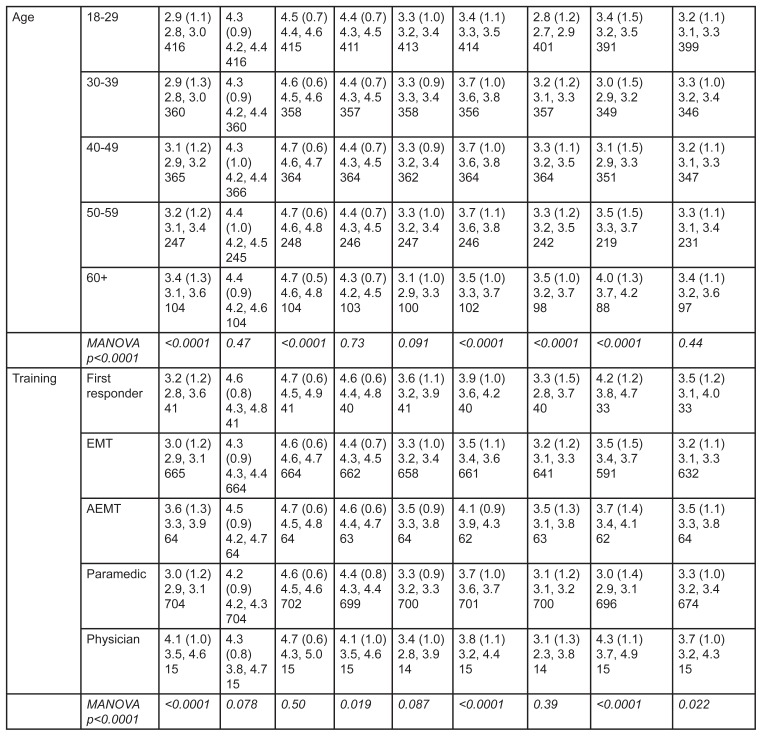

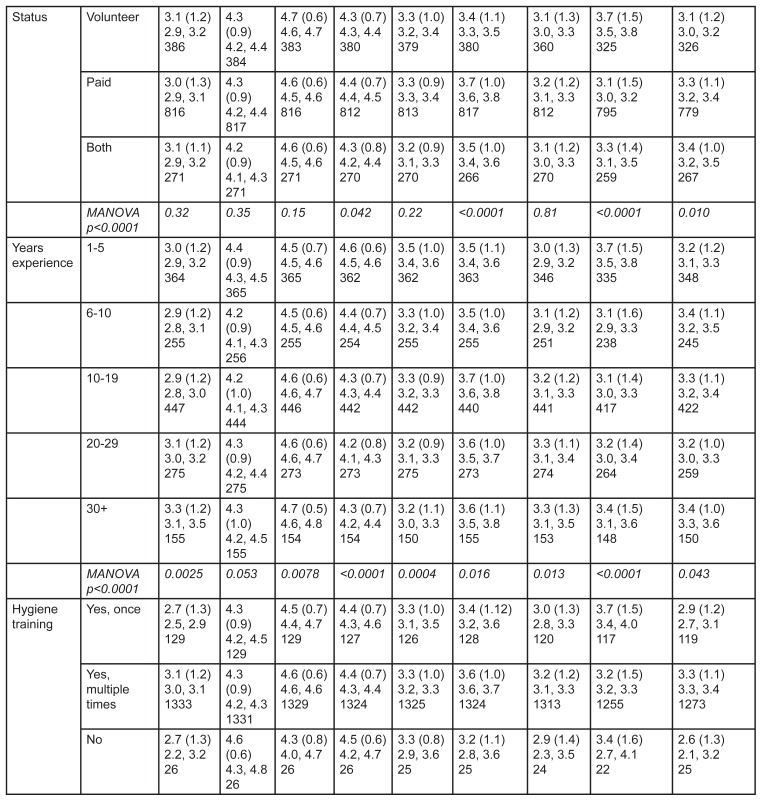

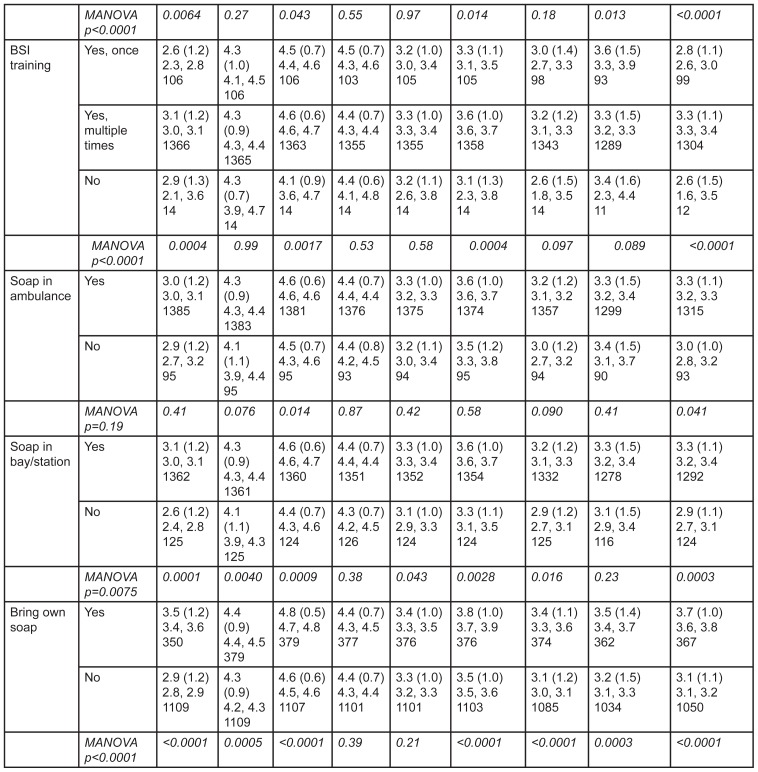
Mean response (standard deviations) stratified by responder characteristics, followed by the 95% confidence interval in the 2nd line, and the absolute number of responses in the 3rd line, per response category. P-values (in italics) are included to test for differences in means of individual responses based on responder characteristic. Multivariate analysis of variance (MANOVA) p-values test whether there is a measurable collective difference over all responses. *MANOVA*, multivariate analysis of variance *EMT*, emergency medical technician; *AEMT*, advanced emergency medical technician; *MANOVA*, multivariate analysis of variance

There were 1,494 survey respondents. Overall frequencies (percentages) as well as means with SDs are presented in Figure 1. Mean responses stratified by participant characteristics are presented in Figure 2, along with p-values for differences between the subgroups, 95% confidence intervals (CI) and the absolute number of responses.

Women reported that they were significantly more likely to clean their hands across almost every category, especially before patient contact and after performing invasive procedures (p<0.0001 for both). The largest gender difference in reported hand hygiene was seen after invasive procedures, with a mean difference on the Likert scale of 0.4 (Males, 95% CI [3.1–3.3]; Females, 95% CI [3.4–3.7]) Overall, women were reportedly more likely to clean their hands in almost every single situation in the survey; absolute differences were small and ranged from 0.1–0.2, and may not be clinically significant.

Increased respondent age was also associated with significantly higher likelihood of reported hand hygiene. Specifically, those 60 years of age or older stated that they were more likely to clean their hands before patient contact, after driving the ambulance and after performing invasive procedures, as opposed to all of the age groups below them (p<0.0001 for all three). The difference on the Likert scale for the three aforementioned situations are 0.5, 0.7, and 0.6 respectively, which suggests a clinical difference.

Level of training and years of experience did not provide many clear relationships regarding hand hygiene practices. However, contrary to most studies performed in the in-hospital environment, EMS physicians were found to clean their hands significantly more than most other groups, specifically in the before patient contact category (largest difference of 1.1, p<0.0001) and after invasive procedures (largest difference of 1.3, p<0.0001). Although the absolute numbers of physician responses was low, it maintained statistical significance.

Paid EMS providers were slightly more likely to report hand hygiene after using equipment, whereas volunteer EMS providers were more likely to report they cleaned their hands after invasive procedures. However, neither of these findings is likely to be clinically significant.

Surprisingly, the presence of hand sanitizer in the ambulance did not make a difference in hand hygiene, except it slightly increased the likelihood of providers cleaning their stethoscopes (p=0.041). However, the presence of hand sanitizer in the ambulance bay was significantly associated with reported increased hand hygiene before patient contact (absolute difference 0.5, p=0.0001) and cleaning the stethoscope (absolute difference 0.4, p=0.0003). This may imply that the availability of cleaning agents just prior to being dispatched may increase hand hygiene compliance. This could be a subtle but important outcome, given our previous finding.

Providers who brought their own hand sanitizer were more likely to clean their hands before patient contact (absolute difference 0.6), after using equipment (absolute difference 0.3), driving (absolute difference 0.3) (p<0.0001 for all three), or performing invasive procedures (absolute difference 0.3, p=0.0003). They also reported they were more likely to clean their own stethoscope (absolute difference 0.6, p< 0.0001).

## DISCUSSION

Our study represents the largest study to date of EMS personnel and hand hygiene. While only a few studies have investigated hand hygiene and infections in the pre-hospital environment, historically, compliance has been poor among healthcare providers. Despite simple solutions like alcohol gels, hand hygiene in the healthcare environment remains a concern.

Our study echoes a previous finding that women were reportedly more likely to clean their hands.^7^

The fact that older respondents reported that they were more likely to wash their hands was an unexpected finding, given the time spent on education for newer healthcare providers about the importance of BSI and the more contemporary, ubiquitous glove use. The recent push by the Centers for Disease Control and Medicare for prevention of infection by hand hygiene may not have had the desired effect on the younger population. Confusingly, other studies have shown that more experienced providers are actually less likely to clean their hands.^8^ Perhaps the providers’ unique setting in EMS has led them to clean their hands more because they perform more frequent procedures. This result may inform the development of future education for hand hygiene.

The increased likelihood of physicians to report they cleaned their hands may both reflect the education physicians receive on the importance of hand hygiene for the prevention of disease transmission both to and from the patient, as well as physicians’ direct interaction with known healthcare-acquired infections. Furthermore, sterile technique procedural education may have played a role. Finally, at least in the United States, their direct participation in field EMS is relatively uncommon outside of the educational arena. The specific situations requiring their involvement may be more likely to be more associated with more ill patients requiring procedures.

Providers who did not experience hand hygiene or BSI training reported they were less likely to clean their stethoscopes than those who had experienced it once or multiple times (p<0.0001). This may be a direct relationship between the amounts of training received and how often providers clean their stethoscopes. This is an important finding, since Merlin, et al. found a significant number of paramedics’ stethoscopes were colonized with MRSA.^8^ An increased rate of stethoscope cleaning could potentially lead to a decreased level of MRSA and other nosocomial infection transmission. This is an area where further research is required to identify a causal rather than associative relationship between infection training and stethoscope cleaning.

Consideration could be given to supplying each EMS provider with personal hand sanitizer, as it appears to be associated with increased reported hand hygiene. This is an inexpensive and potentially positive intervention, and should prompt further research.

We expected the increased availability of sanitizer in the ambulance to make a difference in hand hygiene due to its proximity to the EMS providers and ease of access. In-hospital studies have shown that the placement of gel dispensers has increased the compliance with hand hygiene.^9^–^11^ Therefore, our finding deserves further study on the effects of having hand sanitizer easily available in the ambulance.

There were several concerning findings in this study that require further discussion. Nearly 10% of the respondents either only received blood borne pathogens training or BSI training once or never in their training. This is alarming, given the importance of infection prevention, and when combined with the trend seen in the study, future educational efforts on hand hygiene behavior might have a significant impact.

The reported compliance in situations involving invasive procedures was very concerning. Only 33% reported that they clean their hands after invasive procedures, and 16% reported that the never clean after invasive procedures. Despite the education efforts addressing hygiene, this is a troubling finding, which can potentially increase the risk of disease transmission. In the setting of pre-hospital medicine, with invasive procedures being performed in a non-sterile environment, such as the outdoors, or done in the moving environment of the back of an ambulance, the potential for an exposure significantly increases. Future efforts should be targeted to address this issue, such as supplying personal sanitizer to providers.

In addition, only 56% of the respondents knew that after treating patients with gastrointestinal illnesses, hand washing should occur with soap and water, due to pathogens that are not killed by alcohol-based sanitizers, such as Clostridium difficile and Norwalk virus.^12^ Only 52% of respondents reported that they use gloves with every patient contact. Likewise, only 33% of respondents reported that they cleaned their hands after performing invasive procedures; however, this statistic may be skewed due to the lack of available hand hygiene supplies in the ambulance.

Only 13% reported cleaning their stethoscopes, which is concerning, given the above mentioned study by Merlin et al. about the presence of MRSA on paramedics’ stethoscopes.

Furthermore, only 13% reported cleaning their hands before patient contact. These are all troubling findings, and they identify areas where further education can provide direct results and increase hygiene compliance in these situations.

## LIMITATIONS

We acknowledge several limitations in our study. First, despite our large number of completed surveys, we used a convenience sample so there may have been a selection bias, in that those who chose to respond felt a personal interest, and therefore may have been more likely to over-estimate their hand hygiene practices. Likewise, we used self-report rather than direct observation of cleaning practices which may also have over-estimated the prevalence of hand hygiene. However, since both of these would be expected to skew the results in an over-estimate of actual practice, the areas of concern identified remain striking. Furthermore, we are unable to calculate a response rate due to the method of distribution of the study.

It would be resource intensive and impractical to employ a better methodology to study this topic, such as direct observation. Providing observers to be present on all of the ambulances, or a small selection of ambulances, is time consuming, requires a large amount of resources and is impractical as ambulances do not contain extra space and are cramped to operate in. Likewise, bias may be introduced if the EMS workers realize that they are having their hand washing practices observed, which may lead to a Hawthorne effect.

We note inherent difficulties similar to all retrospective studies in that there may have been recall bias and the findings may only represent an association rather than causal relationship. For example, providers who carry their own hand sanitizer may be particularly attuned to hygiene, and it may not therefore be true that simply issuing sanitizer to all providers will improve hygiene practices for all providers. We also recognize that some of the associations identified may be due to the number of subgroups examined.

Similarly, although many of the results were statistically significant, due to the small absolute difference between the answers, they may not be clinically significant. Also, there may have been geographic bias. While the survey was distributed nationally, we did not know in which area of the country our respondents were practicing. In addition, the response rate per organization is not known.

## CONCLUSION

Our study represents the largest study to date examining the relationship between EMS providers and hand hygiene.

Hand hygiene was reportedly poor overall. Two areas that require further investigation, based on the reportedly poor cleaning, are education on hand hygiene for providers, as well as supplying providers with individual bottles of hand sanitizer. In addition, future education should focus on the importance of cleaning the providers’ stethoscopes, washing hands after any patient contact and the proper technique to clean after exposure to patients with gastrointestinal illness, as these were areas of reportedly poor performance. In an era focused on prevention of disease transmission not only from patient to provider, but provider to patient, this information offers a first step in identifying the problems with EMS provider hygiene, and should prompt more research in this area in order to increase compliance and improve infection rates.

## References

[1] Garus-Pakowska A, Sobala W, Szatko F (2013). Observance of hand washing procedures performed by the medical personnel before patient contact. Part I. Int J Occup Med Environ Health.

[2] Garus-Pakowska A, Sobala W, Szatko F (2013). Observance of hand washing procedures performed by the medical personnel after the patient contact. Part II. Int J Occup Med Environ Health.

[3] Tenias JM, Mayordomo C, Benavent ML (2009). Rev Calidad Asist.

[4] Alter SM, Merlin MA (2011). Nosocomial and community-acquired infection rates of patients treated by prehospital advanced life support compared with other admitted patients. Am J Emerg Med.

[5] Kober P, Labes H, Moller H (2001). Anasthesio Intensivmed Notfallmed Schmerzther.

[6] Merlin MA, Wong ML, Pryor PW (2009). Prevalence of methicillin-resistant Staphylococcus aureus on the stethoscopes of emergency medical services providers. Prehosp Emerg Care.

[7] Hautemaniere A, Cunat L, Diguio N (2010). Factors determining poor practice in alcoholic gel hand rub technique in hospital workers. J Infect Public Health.

[8] Karabay O, Sencan I, Sahin I (2005). Compliance and efficacy of hand rubbing during in-hospital practice. Med Princ Pract.

[9] Zellmer C, Blakney R, Van Hoof S (2015). Impact of sink location on hand hygiene compliance for Clostridium difficile infection. Am J Infect Control.

[10] Eveillard M, Pradelle MT, Lefrancq B (2011). Measurement of hand hygiene compliance and gloving practices in different settings for the elderly considering the location of hand hygiene opportunities during patient care. Am J Infect Control.

[11] Thomas BW, Berg-Copas GM, Vasquez DG (2009). Conspicuous vs customary location of hand hygiene agent dispensers on alcohol-based hand hygiene product usage in an intensive care unit. J Am Osteopath Assoc.

[12] Liu P, Yuen Y, Hsiao HM (2010). Effectiveness of liquid soap and hand sanitizer against Norwalk virus on contaminated hands. Appl Environ Microbiol.

