# Supplementary material for: Hand Washing Practices Among Emergency Medical Services Providers
Source: West J Emerg Med. 2015 Oct 20;16(5):727–35. doi: 10.5811/westjem.2015.7.25917 (PMC4644042; doi:10.5811/westjem.2015.7.25917)
Supplement: Supplementary file 1 [file wjem-16-727-g001.pdf]

Variable	Responses					Mean (SD)
	Male	Female				
Gender	1073 (72%)	421 (28%)				
	18 – 29	30 – 39	40 – 49	50 – 59	60+	
Age	416 (28%)	360 (24%)	367 (25%)	249 (17%)	104 (7%)	
	First Responder	EMT	AEMT	Paramedic	Physician	
Training	41 (3%)	667 (45%)	64 (4%)	705 (47%)	16 (1%)	
	Volunteer	Paid	Both			
Paid/Volunteer	386 (26%)	810 (56%)	272 (18%)			
	1 – 5	6 – 10	10 – 19	20 – 29	30+	
Years of Experience	366 (25%)	256 (17%)	447 (30%)	276 (18%)	155 (10%)	
	Once	Multiple	Never			
BBP Training	129 (9%)	1336 (89%)	26 (2%)			
	Once	Multiple	Never			
BSI Training	106 (7%)	1370 (92%)	14 (1%)			
	Yes	No				
Sanitizer in ambulance	1387 (94%)	95 (6%)				
	Yes	No				
Sanitizer in station	1365 (92%)	126 (8%)				
	Yes	No				
Brings own Sanitizer	380 (25%)	1113 (75%)				
	Never	Rarely	Sometimes	Most of the time	Every time	
Cleans before patient	175 (12%)	394 (26%)	343 (23%)	394 (26%)	190 (13%)	3.0 (1.2)
Cleans after skin contact	21 (1%)	66 (4%)	165 (11%)	430 (29%)	813 (54%)	4.3 (0.9)
Cleans when contact finishes	2 (<1%)	6 (<1%)	76 (5%)	412 (28%)	997 (67%)	4.6 (0.6)
Uses gloves	1 (<1%)	16 (1%)	167 (11%)	522 (35%)	779 (52%)	4.4 (0.7)
Uses gloves with equipment	40 (3%)	252 (17%)	579 (39%)	454 (31%)	159 (11%)	3.3 (1.0)
Cleans after using equipment	28 (2%)	199 (13%)	435 (24%)	492 (33%)	332 (22%)	3.6 (1.0)
Cleans after driving	134 (9%)	341 (23%)	397 (27%)	329 (22%)	264 (18%)	3.2 (1.2)

**Cleans after invasive procedures**	228 (16%)	273 (19%)	227 (16%)	218 (16%)	456 (33%)	3.3 (1.5)
**Cleans stethoscope**	99 (7%)	223 (16%)	490 (34%)	426 (30%)	186 (13%)	3.3 (1.1)
	**Soap**	**Sanitizer**	**Do not clean after**			
**Cleans after hands visibly contaminated**	1258 (84%)	239 (16%)				
	**Soap**	**Sanitizer**	**No preference**			
**Soap or Sanitizer preference**	1059 (71%)	255 (17%)	185 (12%)			
	**Yes**	**No**	**I don't know**			
**Must use soap with GI illness**	839 (56%)	534 (36%)	124 (8%)			
