# Supplementary material for: Hand Washing Practices Among Emergency Medical Services Providers
Source: West J Emerg Med. 2015 Oct 20;16(5):727–35. doi: 10.5811/westjem.2015.7.25917 (PMC4644042; doi:10.5811/westjem.2015.7.25917)
Supplement: Supplementary file 2 [file wjem-16-727-g002.pdf]

		Response						
Responder Characteristic		Clean before Contact	Clean after Skin	Clean after over	Gloves	Gloves supplies	Clean after equipment	Clean Driving
Gender	Male	2.9 (1.2)<br>2.9, 3.0<br>1071	4.3 (0.9)<br>4.2, 4.3<br>1071	4.6 (0.6)<br>4.5, 4.6<br>1068	4.4 (0.8)<br>4.3, 4.4<br>1064	3.3 (1.0)<br>3.2, 3.3<br>1064	3.6 (1.0)<br>3.5, 3.6<br>1065	3.1 (1.2<br>3.0, 3.2<br>1060
	Female	3.2 (1.2)<br>3.1, 3.3<br>419	4.4 (0.9)<br>4.3, 4.5<br>418	4.7 (0.5)<br>4.7, 4.8<br>419	4.4 (0.7)<br>4.4, 4.5<br>415	3.4 (0.9)<br>3.3, 3.5<br>415	3.7 (1.1)<br>3.6, 3.8<br>415	3.3 (1.3<br>3.2, 3.4<br>399
	MANOVA p<0.0001	*<0.0001*	*0.0038*	*<0.0001*	*0.29*	*0.052*	*0.22*	*0.0057*
Age	18-29	2.9 (1.1)<br>2.8, 3.0<br>416	4.3 (0.9)<br>4.2, 4.4<br>416	4.5 (0.7)<br>4.4, 4.6<br>415	4.4 (0.7)<br>4.3, 4.5<br>411	3.3 (1.0)<br>3.2, 3.4<br>413	3.4 (1.1)<br>3.3, 3.5<br>414	2.8 (1.2<br>2.7, 2.9<br>401
	30-39	2.9 (1.3)<br>2.8, 3.0<br>360	4.3 (0.9)<br>4.2, 4.4<br>360	4.6 (0.6)<br>4.5, 4.6<br>358	4.4 (0.7)<br>4.3, 4.5<br>357	3.3 (0.9)<br>3.3, 3.4<br>358	3.7 (1.0)<br>3.6, 3.8<br>356	3.2 (1.2<br>3.1, 3.3<br>357
	40-49	3.1 (1.2)<br>2.9, 3.2<br>365	4.3 (1.0)<br>4.2, 4.4<br>366	4.7 (0.6)<br>4.6, 4.7<br>364	4.4 (0.7)<br>4.3, 4.5<br>364	3.3 (0.9)<br>3.2, 3.4<br>362	3.7 (1.0)<br>3.6, 3.8<br>364	3.3 (1.1<br>3.2, 3.5<br>364
	50-59	3.2 (1.2)<br>3.1, 3.4<br>247	4.4 (1.0)<br>4.2, 4.5<br>245	4.7 (0.6)<br>4.6, 4.8<br>248	4.4 (0.7)<br>4.3, 4.5<br>246	3.3 (1.0)<br>3.2, 3.4<br>247	3.7 (1.1)<br>3.6, 3.8<br>246	3.3 (1.2<br>3.2, 3.5<br>242
	60+	3.4 (1.3)<br>3.1, 3.6<br>104	4.4 (0.9)<br>4.2, 4.6<br>104	4.7 (0.5)<br>4.6, 4.8<br>104	4.3 (0.7)<br>4.2, 4.5<br>103	3.1 (1.0)<br>2.9, 3.3<br>100	3.5 (1.0)<br>3.3, 3.7<br>102	3.5 (1.0<br>3.2, 3.7<br>98
	MANOVA p<0.0001	*<0.0001*	*0.47*	*<0.0001*	*0.73*	*0.091*	*<0.0001*	*<0.000*
Training	First Responder	3.2 (1.2)<br>2.8, 3.6<br>41	4.6 (0.8)<br>4.3, 4.8<br>41	4.7 (0.6)<br>4.5, 4.9<br>41	4.6 (0.6)<br>4.4, 4.8<br>40	3.6 (1.1)<br>3.2, 3.9<br>41	3.9 (1.0)<br>3.6, 4.2<br>40	3.3 (1.5<br>2.8, 3.7<br>40
	EMT	3.0 (1.2)<br>2.9, 3.1<br>665	4.3 (0.9)<br>4.3, 4.4<br>664	4.6 (0.6)<br>4.6, 4.7<br>664	4.4 (0.7)<br>4.3, 4.5<br>662	3.3 (1.0)<br>3.2, 3.4<br>658	3.5 (1.1)<br>3.4, 3.6<br>661	3.2 (1.2<br>3.1, 3.3<br>641
	AEMT	3.6 (1.3)<br>3.3, 3.9<br>64	4.5 (0.9)<br>4.2, 4.7<br>64	4.7 (0.6)<br>4.5, 4.8<br>64	4.6 (0.6)<br>4.4, 4.7<br>63	3.5 (0.9)<br>3.3, 3.8<br>64	4.1 (0.9)<br>3.9, 4.3<br>62	3.5 (1.3<br>3.1, 3.8<br>63
	Paramedic	3.0 (1.2)<br>2.9, 3.1<br>704	4.2 (0.9)<br>4.2, 4.3<br>704	4.6 (0.6)<br>4.5, 4.6<br>702	4.4 (0.8)<br>4.3, 4.4<br>699	3.3 (0.9)<br>3.2, 3.3<br>700	3.7 (1.0)<br>3.6, 3.7<br>701	3.1 (1.2<br>3.1, 3.2<br>700
	Physician	4.1 (1.0)<br>3.5, 4.6<br>15	4.3 (0.8)<br>3.8, 4.7<br>15	4.7 (0.6)<br>4.3, 5.0<br>15	4.1 (1.0)<br>3.5, 4.6<br>15	3.4 (1.0)<br>2.8, 3.9<br>14	3.8 (1.1)<br>3.2, 4.4<br>15	3.1 (1.3<br>2.3, 3.8<br>14
	MANOVA p<0.0001	*<0.0001*	*0.078*	*0.50*	*0.019*	*0.087*	*<0.0001*	*0.39*
Status	Volunteer	3.1 (1.2)<br>2.9, 3.2<br>386	4.3 (0.9)<br>4.2, 4.4<br>384	4.7 (0.6)<br>4.6, 4.7<br>383	4.3 (0.7)<br>4.3, 4.4<br>380	3.3 (1.0)<br>3.2, 3.4<br>379	3.4 (1.1)<br>3.3, 3.5<br>380	3.1 (1.3<br>3.0, 3.3<br>360
	Paid	3.0 (1.3)<br>2.9, 3.1<br>816	4.3 (0.9)<br>4.2, 4.4<br>817	4.6 (0.6)<br>4.5, 4.6<br>816	4.4 (0.7)<br>4.4, 4.5<br>812	3.3 (0.9)<br>3.3, 3.4<br>813	3.7 (1.0)<br>3.6, 3.8<br>817	3.2 (1.2<br>3.1, 3.3<br>812
	Both	3.1 (1.1)<br>2.9, 3.2<br>271	4.2 (0.9)<br>4.1, 4.3<br>271	4.6 (0.6)<br>4.5, 4.6<br>271	4.3 (0.8)<br>4.2, 4.4<br>270	3.2 (0.9)<br>3.1, 3.3<br>270	3.5 (1.0)<br>3.4, 3.6<br>266	3.1 (1.2<br>3.0, 3.3<br>270
	MANOVA p<0.0001	*0.32*	*0.35*	*0.15*	*0.042*	*0.22*	*<0.0001*	*0.81*
Years Experience	1-5	3.0 (1.2)<br>2.9, 3.2<br>364	4.4 (0.9)<br>4.3, 4.5<br>365	4.5 (0.7)<br>4.5, 4.6<br>365	4.6 (0.6)<br>4.5, 4.6<br>362	3.5 (1.0)<br>3.4, 3.6<br>362	3.5 (1.1)<br>3.4, 3.6<br>363	3.0 (1.3<br>2.9, 3.2<br>346
	6-10	2.9 (1.2)<br>2.8, 3.1<br>255	4.2 (0.9)<br>4.1, 4.3<br>256	4.5 (0.6)<br>4.5, 4.6<br>255	4.4 (0.7)<br>4.4, 4.5<br>254	3.3 (1.0)<br>3.2, 3.4<br>255	3.5 (1.0)<br>3.4, 3.6<br>255	3.1 (1.2<br>2.9, 3.2<br>251
	10-19	2.9 (1.2)<br>2.8, 3.0	4.2 (1.0)<br>4.1, 4.3	4.6 (0.6)<br>4.6, 4.7	4.3 (0.7)<br>4.3, 4.4	3.3 (0.9)<br>3.2, 3.3	3.7 (1.0)<br>3.6, 3.8	3.2 (1.2<br>3.1, 3.3

		447	444	446	442	442	440	441
	20-29	3.1 (1.2)<br>3.0, 3.2<br>275	4.3 (0.9)<br>4.2, 4.4<br>275	4.6 (0.6)<br>4.6, 4.7<br>273	4.2 (0.8)<br>4.1, 4.3<br>273	3.2 (0.9)<br>3.1, 3.3<br>275	3.6 (1.0)<br>3.5, 3.7<br>273	3.3 (1.1<br>3.1, 3.4<br>274
	30+	3.3 (1.2)<br>3.1, 3.5<br>155	4.3 (1.0)<br>4.2, 4.5<br>155	4.7 (0.5)<br>4.6, 4.8<br>154	4.3 (0.7)<br>4.2, 4.4<br>154	3.2 (1.1)<br>3.0, 3.3<br>150	3.6 (1.1)<br>3.5, 3.8<br>155	3.3 (1.3<br>3.1, 3.5<br>153
	MANOVA p<0.0001	*0.0025*	*0.053*	*0.0078*	*<0.0001*	*0.0004*	*0.016*	*0.013*
Hygiene Training	Yes, once	2.7 (1.3)<br>2.5, 2.9<br>129	4.3 (0.9)<br>4.2, 4.5<br>129	4.5 (0.7)<br>4.4, 4.7<br>129	4.4 (0.7)<br>4.3, 4.6<br>127	3.3 (1.0)<br>3.1, 3.5<br>126	3.4 (1.12)<br>3.2, 3.6<br>128	3.0 (1.3<br>2.8, 3.3<br>120
	Yes, multiple times	3.1 (1.2)<br>3.0, 3.1<br>1333	4.3 (0.9)<br>4.2, 4.3<br>1331	4.6 (0.6)<br>4.6, 4.6<br>1329	4.4 (0.7)<br>4.3, 4.4<br>1324	3.3 (1.0)<br>3.2, 3.3<br>1325	3.6 (1.0)<br>3.6, 3.7<br>1324	3.2 (1.2<br>3.1, 3.3<br>1313
	No	2.7 (1.3)<br>2.2, 3.2<br>26	4.6 (0.6)<br>4.3, 4.8<br>26	4.3 (0.8)<br>4.0, 4.7<br>26	4.5 (0.6)<br>4.2, 4.7<br>26	3.3 (0.8)<br>2.9, 3.6<br>25	3.2 (1.1)<br>2.8, 3.6<br>25	2.9 (1.4<br>2.3, 3.5<br>24
	MANOVA p<0.0001	*0.0064*	*0.27*	*0.043*	*0.55*	*0.97*	*0.014*	*0.18*
BSI training	Yes, once	2.6 (1.2)<br>2.3, 2.8<br>106	4.3 (1.0)<br>4.1, 4.5<br>106	4.5 (0.7)<br>4.4, 4.6<br>106	4.5 (0.7)<br>4.3, 4.6<br>103	3.2 (1.0)<br>3.0, 3.4<br>105	3.3 (1.1)<br>3.1, 3.5<br>105	3.0 (1.4<br>2.7, 3.3<br>98
	Yes, multiple times	3.1 (1.2)<br>3.0, 3.1<br>1366	4.3 (0.9)<br>4.3, 4.4<br>1365	4.6 (0.6)<br>4.6, 4.7<br>1363	4.4 (0.7)<br>4.3, 4.4<br>1355	3.3 (1.0)<br>3.3, 3.4<br>1355	3.6 (1.0)<br>3.6, 3.7<br>1358	3.2 (1.2<br>3.1, 3.3<br>1343
	No	2.9 (1.3)<br>2.1, 3.6<br>14	4.3 (0.7)<br>3.9, 4.7<br>14	4.1 (0.9)<br>3.6, 4.7<br>14	4.4 (0.6)<br>4.1, 4.8<br>14	3.2 (1.1)<br>2.6, 3.8<br>14	3.1 (1.3)<br>2.3, 3.8<br>14	2.6 (1.5<br>1.8, 3.5<br>14
	MANOVA p<0.0001	*0.0004*	*0.99*	*0.0017*	*0.53*	*0.58*	*0.0004*	*0.097*
Soap in ambulance	Yes	3.0 (1.2)<br>3.0, 3.1<br>1385	4.3 (0.9)<br>4.3, 4.4<br>1383	4.6 (0.6)<br>4.6, 4.6<br>1381	4.4 (0.7)<br>4.4, 4.4<br>1376	3.3 (1.0)<br>3.2, 3.3<br>1375	3.6 (1.0)<br>3.6, 3.7<br>1374	3.2 (1.2<br>3.1, 3.2<br>1357
	No	2.9 (1.2)<br>2.7, 3.2<br>95	4.1 (1.1)<br>3.9, 4.4<br>95	4.5 (0.7)<br>4.3, 4.6<br>95	4.4 (0.8)<br>4.2, 4.5<br>93	3.2 (1.1)<br>3.0, 3.4<br>94	3.5 (1.2)<br>3.3, 3.8<br>95	3.0 (1.2<br>2.7, 3.2<br>94
	MANOVA p=0.19	*0.41*	*0.076*	*0.014*	*0.87*	*0.42*	*0.58*	*0.090*
Soap in bay/station	Yes	3.1 (1.2)<br>3.0, 3.1<br>1362	4.3 (0.9)<br>4.3, 4.4<br>1361	4.6 (0.6)<br>4.6, 4.7<br>1360	4.4 (0.7)<br>4.4, 4.4<br>1351	3.3 (1.0)<br>3.3, 3.4<br>1352	3.6 (1.0)<br>3.6, 3.7<br>1354	3.2 (1.2<br>3.1, 3.3<br>1332
	No	2.6 (1.2)<br>2.4, 2.8<br>125	4.1 (1.1)<br>3.9, 4.3<br>125	4.4 (0.7)<br>4.3, 4.6<br>124	4.3 (0.7)<br>4.2, 4.5<br>126	3.1 (1.0)<br>2.9, 3.3<br>124	3.3 (1.1)<br>3.1, 3.5<br>124	2.9 (1.2<br>2.7, 3.1<br>125
	MANOVA p=0.0075	*0.0001*	*0.0040*	*0.0009*	*0.38*	*0.043*	*0.0028*	*0.016*
Bring own soap	Yes	3.5 (1.2)<br>3.4, 3.6<br>350	4.4 (0.9)<br>4.4, 4.5<br>379	4.8 (0.5)<br>4.7, 4.8<br>379	4.4 (0.7)<br>4.3, 4.5<br>377	3.4 (1.0)<br>3.3, 3.5<br>376	3.8 (1.0)<br>3.7, 3.9<br>376	3.4 (1.1<br>3.3, 3.6<br>374
	No	2.9 (1.2)<br>2.8, 2.9<br>1109	4.3 (0.9)<br>4.2, 4.3<br>1109	4.6 (0.6)<br>4.5, 4.6<br>1107	4.4 (0.7)<br>4.3, 4.4<br>1101	3.3 (1.0)<br>3.2, 3.3<br>1101	3.5 (1.0)<br>3.5, 3.6<br>1103	3.1 (1.2<br>3.0, 3.1<br>1085
	MANOVA p<0.0001	*<0.0001*	*0.0005*	*<0.0001*	*0.39*	*0.21*	*<0.0001*	*<0.000*
